# Activation of Akt by Advanced Glycation End Products (AGEs): Involvement of IGF-1 Receptor and Caveolin-1

**DOI:** 10.1371/journal.pone.0058100

**Published:** 2013-03-05

**Authors:** Su-Jung Yang, Chen-Yu Chen, Geen-Dong Chang, Hui-Chin Wen, Ching-Yu Chen, Shi-Chuan Chang, Jyh-Fei Liao, Chung-Ho Chang

**Affiliations:** 1 Department and Institute of Pharmacology, School of Medicine, National Yang-Ming University, Taipei, Taiwan, Republic of China; 2 Institute of Cellular and System Medicine, National Health Research Institutes, Zhunan, Miaoli, Taiwan, Republic of China; 3 Graduate Institute of Biochemical Sciences, National Taiwan University, Taipei, Taiwan, Republic of China; 4 Department of Family Medicine, National Taiwan University Hospital and College of Medicine, National Taiwan University, Taipei, Taiwan; 5 Division of Geriatric Research, Institute of Population Health Sciences, National Health Research Institutes, Zhunan, Miaoli, Taiwan, Republic of China; 6 Chest Department, Taipei Veterans General Hospital, Institute of Emergency and Critical Care Medicine, National Yang-Ming University, Taipei, Taiwan, Republic of China; 7 Ph.D. Program for Aging, College of Medicine, China Medical University, Taichung, Taiwan, Republic of China; AMS Biotechnology, United Kingdom

## Abstract

Diabetes is characterized by chronic hyperglycemia, which in turn facilitates the formation of advanced glycation end products (AGEs). AGEs activate signaling proteins such as Src, Akt and ERK1/2. However, the mechanisms by which AGEs activate these kinases remain unclear. We examined the effect of AGEs on Akt activation in 3T3-L1 preadipocytes. Addition of AGEs to 3T3-L1 cells activated Akt in a dose- and time-dependent manner. The AGEs-stimulated Akt activation was blocked by a PI3-kinase inhibitor LY 294002, Src inhibitor PP2, an antioxidant NAC, superoxide scavenger Tiron, or nicotinamide adenine dinucleotide phosphate (NAD(P)H) oxidase inhibitor DPI, suggesting the involvement of Src and NAD(P)H oxidase in the activation of PI3-kinase-Akt pathway by AGEs. AGEs-stimulated Src tyrosine phosphorylation was inhibited by NAC, suggesting that Src is downstream of NAD(P)H oxidase. The AGEs-stimulated Akt activity was sensitive to Insulin-like growth factor 1 receptor (IGF-1R) kinase inhibitor AG1024. Furthermore, AGEs induced phosphorylation of IGF-1 receptorβsubunit (IGF-1Rβ) on Tyr1135/1136, which was sensitive to PP2, indicating that AGEs stimulate Akt activity by transactivating IGF-1 receptor. In addition, the AGEs-stimulated Akt activation was attenuated by β-methylcyclodextrin that abolishes the structure of caveolae, and by lowering caveolin-1 (Cav-1) levels with siRNAs. Furthermore, addition of AGEs enhanced the interaction of phospho-Cav-1 with IGF-1Rβ and transfection of 3T3-L1 cells with Cav-1 Y14F mutants inhibited the activation of Akt by AGEs. These results suggest that AGEs activate NAD(P)H oxidase and Src which in turn phosphorylates IGF-1 receptor and Cav-1 leading to activation of IGF-1 receptor and the downstream Akt in 3T3-L1 cells. AGEs treatment promoted the differentiation of 3T3-L1 preadipocytes and addition of AG1024, LY 294002 or Akt inhibitor attenuated the promoting effect of AGEs on adipogenesis, suggesting that IGF-1 receptor, PI3-Kinase and Akt are involved in the facilitation of adipogenesis by AGEs.

## Introduction

Glucose and other reducing sugars can react non-enzymatically with the amino groups of proteins and lipids to form Schiff bases. The Schiff bases are slowly rearranged to form Amadori products which then undergo further rearrangements, oxidation, dehydration and condensation resulting in compounds called advanced glycation end products (AGEs). AGEs are formed in normal physiological condition. The formation and accumulation of AGEs are accelerated in tissues from aged individuals and diabetic patients [1]–[6].

AGEs exert their cellular functions via the interaction with their specific receptor, the receptor for advanced glycation end products (RAGE) [7]. Binding of AGEs to RAGE activates a variety of signaling proteins and downstream transcription factors including Src, NAD(P)H oxidase, Ras/ERK1/2, PI3K/PDK1/Akt, p38 MAPK, NF-κB, and AP1 [1], [3], [5]. Activation of RAGE by AGEs stimulates the production of reactive oxygen species (ROS) by NAD(P)H oxidase or mitochondria in several cell types [8]–[13]. It has been shown that activation of PI3K by AGEs is dependent on the generation of ROS in mesangial cells [10]. However, little is known about the proximal signaling events downstream of RAGE.

Caveolae are membrane regions enriched in cholesterol and glycosphingolipids. The major proteins in caveolae are caveolins which serve as structural elements of caveolae. The mammalian caveolin gene family consists of caveolin-1, -2 and -3. Caveolin-1 (Cav-1) is ubiquitously expressed. Caveolin-2 is co-expressed with Cav-1, whereas caveolin-3 is exclusively expressed in skeletal, cardiac and smooth muscle cells [14]. Caveolins function as scaffolding proteins which recruit numerous signaling molecules to the plasma membrane and regulate their activity. For instance, RAGE, EGF receptor, insulin receptor, IGF-I receptor, Src, PKA, PKC, Akt, ERK1/2, p38 MAPK and PI3-kinase are localized in caveolae [for review, see 14–18]. Therefore, caveolae represent areas in which signaling proteins and their downstream effectors are substantially enriched.

Adipose tissue comprises of adipocytes, preadipocytes and other cell types. All cell types in adipose tissue are constantly exposed to AGEs that are generated even in the normal glucose condition. 3T3-L1 cells, derived from dissociated near term Swiss 3T3 mouse embryos, are a widely used model system for preadipocytes [19]. In this study, we investigate the mechanisms by which AGEs activate Akt in 3T3-L1 preadipocytes. Since RAGE and several signaling proteins are localized in caveolae and caveolins regulate a variety of signaling pathways, we also examined the involvement of Cav-1 in RAGE-mediated Akt activation in 3T3-L1 cells. Our results showed that NAD(P)H oxidase, Src and IGF-1 receptor transactivation are involved in the activation of Akt by AGEs. 3T3-L1 cells can be induced to differentiate into adipocytes by IBMX, dexamethasone, and insulin/IGF-1. Since AGEs transactivate IGF-1 receptor, we further examined whether AGEs affect the differentiation of 3T3-L1 cells.

## Materials and Methods

### Antibodies and Chemicals

Antibodies against phospho-Src, Src, phospho-Akt, Akt, PDK1, Cav-1, IRS-1, aP2, IGF-1R and phosphor-IGF-1R (at Y1131 and Y1135/1136) antibodies were purchased from Cell Signaling (Beverly, MA). Phospho-tyrosine antibodies were obtained from Upstate Biochemical Inc. (Lake Placid, NY). Phospho-(Tyr-14)-Cav-1 antibody was purchased from BD Biosciences. RAGE, PPARγ and C/EBPα antibodies were obtained from Santa Cruz Biotechnology (Santa Cruz, CA). Antibody against GAPDH was purchased from Zymed (Carlsbad, CA, USA). Phospho-(S241)-PDK1 antibody was obtained from BD Pharmingen (San Jose, CA). Immobilon-P membrane was purchased from Millipore (Bedford, MA). AG1024 and PP2 were purchased from Calbiochem (San Diego, CA). Cav-1 siRNAs were obtained from Dharmacon Inc. (Lafayette, CO). The BCA protein assay kit and the enhanced chemiluminescence (ECL) detection system kit were purchased from Perkin Elmer Life Sciences, Inc**.** (Waltham, MA). CM-H2DCFDA was obtained from Molecular Probes Inc. (Eugene, OR). Actin antibody and other chemicals were purchased from Sigma (St. Louis, MO).

### Preparation of Advanced Glycation End Product (AGEs)

AGEs were prepared as described by Hamada Y. et al. [20]. Briefly, bovine serum albumin (BSA) (10 mg/ml) was incubated with 33 mM glyceraldehyde and 100 U/ml penicillin/streptomycin in PBS, pH 7.4 at 37°C for 3 days in the dark. After 3 days incubation, AGEs were dialyzed in PBS (pH 7.4) at 4°C for 2 days, and then sterilized with 0.22 µm filter.

### Cell Culture

3T3-L1 preadipocytes (CL-173; ATCC,USA) were maintained at 37°C in high glucose Dulbecco’s modified Eagle’s medium (DMEM) with 50 units/ml penicillin, 50 µg/ml streptomycin, and 10% calf serum in 5% CO_2_ environment. Near confluent 3T3-L1 cells were incubated with serum-free media for 18 h to arrest and synchronize the cell growth. After this time period, the cells were treated with and without an antioxidant N-acetyl-cysteine, superoxide scavenger Tiron, NADPH oxidase inhibitor diphenyleneiodonium chloride, specific Src family kinase inhibitor PP2, IGF-1 receptor kinase inhibitor AG1024, PI3K inhibitor LY294002, or RAGE antibody for the indicated time before the addition of 100 µg/m AGEs.

### Measurement of Reactive Oxygen Species

Levels of intracellular reactive oxygen species (ROS) were assessed spectro-fluorimetrically by the oxidation of a specific probe CM-H_2_DCFDA. 3T3-L1 cells were seeded in a 6 wells plate and serum-starved for 18 h. The cells were exposed to 100 µg/ml AGEs for 15 min in the serum-free medium. After stimulation, cells were washed twice with Hank’s Buffered Salt Solution (HBSS). Cells were trypsinized and re-suspended in 1 ml HBSS. The cell suspension was loaded with 20 µM CM-H_2_DCFDA for 30 min at 37°C. After incubation, the cells were washed two times with HBSS. The suspension was added to 96 wells dark plates for detection. The production of ROS was determined by a fluorescence reader (excitation/emission: 485/520 nm).

### Generation of a Cav-1 Y14F Stable Cell Line

Mouse Cav-1 cDNA was subcloned into the pcDNA3.1 plasmid (Invitrogen). The Y14F mutant of Cav-1 construct was generated using QuickChange® site-directed mutagenesis kit (Stratagene, La Jolla, CA, USA) [21] with the following primer pairs (synthesized by the Core Instrument Center at the National Health Research Institute), 5′-AGGGACATCTCTTCACTGTTCCCAT-3′ and 5′-ATGGGAACAGTGAAGAGATGTCCCT-3′. For transfection, a density of 2×10^5^ 3T3-L1 cells were plated into 60 mm dishes and maintained in DMEM containing 10% FBS, 3.7 g/L NaHCO3, 5.9575 g/L HEPES, and 100 U/ml penicillin/streptomycin at 37°C, 5% CO2. After 24 hours, cells were transfected by Lipofectamine 2000 (Invitrogen) with 2.5 µg of plasmids: pcDNA3.1-Cav-1 mutant (Cav-1 Y14F) and vector pcDNA3.1 as the positive control. The following day, cells were split and selected in 500 µg/ml neomycin to generate pcDNA3.1-Cav-1 mutant (Cav-1 Y14F) and pcDNA3.1 stable cell lines.

### Western Blot Analysis

3T3-L1 cells were starved for 18 h in DMEM, and then stimulated with 100 µg/ml AGEs for 15 min at 37°C. Cells were washed with ice-cold PBS and lysed in RIPA buffer [20 mM Tris-HCl (pH 8.0), 10% glycerol, 150 mM NaCl, 1% Nonidet P-40 (NP-40) and 0.42% NaF] containing protease inhibitors (5 µg/mL aprotinin, 5 µg/mL leupetin, 1 mM Na3VO4, 1 mM phenylmethylsulfonyl fluoride, and 20 mM NaF). Cell lysates were centrifuged at 12000 rpm for 15 min to remove insoluble materials. Western blotting was performed as previously described [21], [22]. The following antibodies were used: Akt, phospho-Akt (at serine 473 and threonine 308), PDK1, phosphor-PDK1 (at Ser 241), Cav-1, phospho-Cavolin-1 (at tyrosine 14), IGF-1R, phospho-IGF-1Rβ (at tyrosine 1131 and tyrosine 1135/1136), IRS-1, phospho-IRS-1, Src, phospho-Src (at tyrosine 416), phospho-tyrosine, PPARγ, aP2, GAPDH or β-actin.

### Co-immunoprecipitation

3T3-L1 cell lysates were prepared as described under Western blot analysis and subjected to immunoprecipitation with IGF-1Rβ antibodies or control mouse IgG (Sigma-Aldrich) using the immunoprecipitation kit Catch and Release® v2.0 (Upstate Signaling solution) following the manufacturer’s instruction. The effluent was analyzed by Western blot analysis. Briefly, 500 µg of cell lysates, 5 µg of anti-IGF-1Rβ antibody, IRS-1 or control mouse IgG (Sigma-Aldrich) and 10 µl of antibody capture affinity ligand were mixed and placed in a catch and release® v2.0 spin column containing 0.5 ml of prepacked IP capture resin. After end-over-end shaking for 12 h in 4°C, the column was centrifuged, washed 3 times and then eluted with 70 µl of the elution buffer. The effluent was analyzed by Western blot analysis with Akt, phospho-Akt (at serine 473 and threonine 308), Cav-1, phospho-Cavolin-1 (at tyrosine 14), IGF-1R, phospho-IGF-1Rβ (at tyrosine 1131 and tyrosine 1135/1136), IRS-1, phospho-IRS-1, Src, or phospho-Src (at tyrosine 416).

### Adipogenic Differentiation

For the induction of differentiation, at 2 days post-confluence, 3T3-L1 preadipocytes were incubated with DMEM containing 0.5 mM IBMX, 1 µM dexamethasone, 1.7 µM insulin, and 10% FBS at 37°C, 5% CO_2_ for 72 hr. The induction medium was replaced with culture medium containing 1.7 µM insulin on day 3. Since day 5, the fresh culture medium was replaced every other day. The oil droplet contents were quantified by measuring the absorbance of oil red O at OD510 nm.

### Oil Red O Staining

Differentiated 3T3-L1 cells were washed three times with PBS, and then fixed with 4% paraformaldehyde at room temperature. After 10 min, the fix solution was removed and cells were washed three times with PBS. The 0.35% oil red O stock solution were diluted with ddH_2_O (in 3∶2 ratio) and incubated with cells at 37°C for 1 hr. Stained cells were washed with PBS and visualized.

### Glycerol-3-phosphate Dehydrogenase (GPDH) Assay

On day 8 after the induction of differentiation, 3T3-L1 cells were washed twice with PBS, incubated, and scraped with the homogenization buffer which contains 0.25 M sucrose, 1 mM EDTA, 1 mM dithiothreitol, and 5 mM Tris-HCl (pH 7.6). Homogenates were sonicated on ice for 5 sec three times, and centrifuged (12000 rpm) for 10 min at 4°C. The protein amounts were determined with a BCA protein assay kit following the manufacture’s instruction. The amounts of OD_340_ were determined at 30°C with 30 sec intervals for 8 min. Enzyme activities (U/µg protein/min) were obtained from the decline of absorbance within 8 min [23].

### Statistical Analyses

The experimental results are expressed as the mean values ± SEM and are accompanied by the number of observations. Data were assessed by the Student’s *t*-test method. A *p* value of less than 0.05 was considered statistically significant.

## Results

### AGEs-induced Phosphorylation of Akt is Mediated by Activation of PI3-Kinase in 3T3-L1 Cells

**Figure 1:**
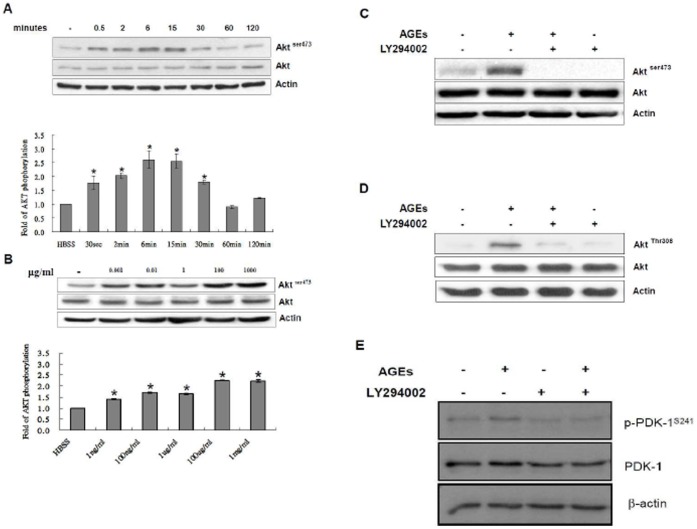
Time- and dose-dependent activation of Akt by AGEs in 3T3-L1 cells. (**A**) Serum-starved quiescent 3T3-L1 cells were exposed to AGEs (100 µg/ml) for the indicated times. (**B**) Serum-starved quiescent 3T3-L1 cells were exposed to various concentrations of AGEs for 15 min. (**C**) Serum-starved quiescent 3T3-L1 cells were pretreated with 15 µM LY294002 for 30 min, and then exposed to 100 µg/ml AGEs for 15 min. Total cell lysates were immunoblotted with antibodies recognizing phospho-Akt (Akt^ser473^), Akt or actin. (**D**) Serum-starved quiescent 3T3-L1 cells were pretreated with 15 µM LY294002 for 30 min, and then exposed to 100 µg/ml AGEs for 15 min. Total cell lysates were immunoblotted with antibodies recognizing phospho-Akt (Akt^Thr308^), Akt or actin. (**E**) Serum-starved quiescent 3T3-L1 cells were pretreated with 15 µM LY294002 for 30 min, and then exposed to 100 µg/ml AGEs for 15 min. Total cell lysates were immunoblotted with antibodies recognizing phospho-PDK1 (PDK1^Ser241^), PDK1 or actin. Data are representative of three independent experiments yielding similar results. *, statistically significant differences (*, *P <*0.05 *versus* control).

AGEs have been shown to activate PI3-K and Akt in neutrophils and renal mesangial cells [10], [24]. However, the molecular mechanisms by which AGEs activate Akt remain unclear. The effects of AGEs on the PI3-kinase-Akt pathway in 3T3-L1 cells have not been reported, and were examined in this study. As shown in Fig. 1A and 1*B*, AGEs activated Akt in a time-dependent and dose-dependent manner. Pre-treatment of 3T3-L1 cells with a PI3-kinase inhibitor LY294002 (15 µM) for 30 min completely blocked the activation of Akt induced by AGEs (Fig. 1C and 1D). AGEs also activated PDK1 and PDK1 activation was completely blocked by LY294002 (Fig. 1E). These results suggest that AGEs activate Akt via PI3-kinase and PDK1 in 3T3-L1 cells.

### ROS are Required for AGEs-induced Akt Phosphorylation in 3T3-L1 Cells

**Figure 2:**
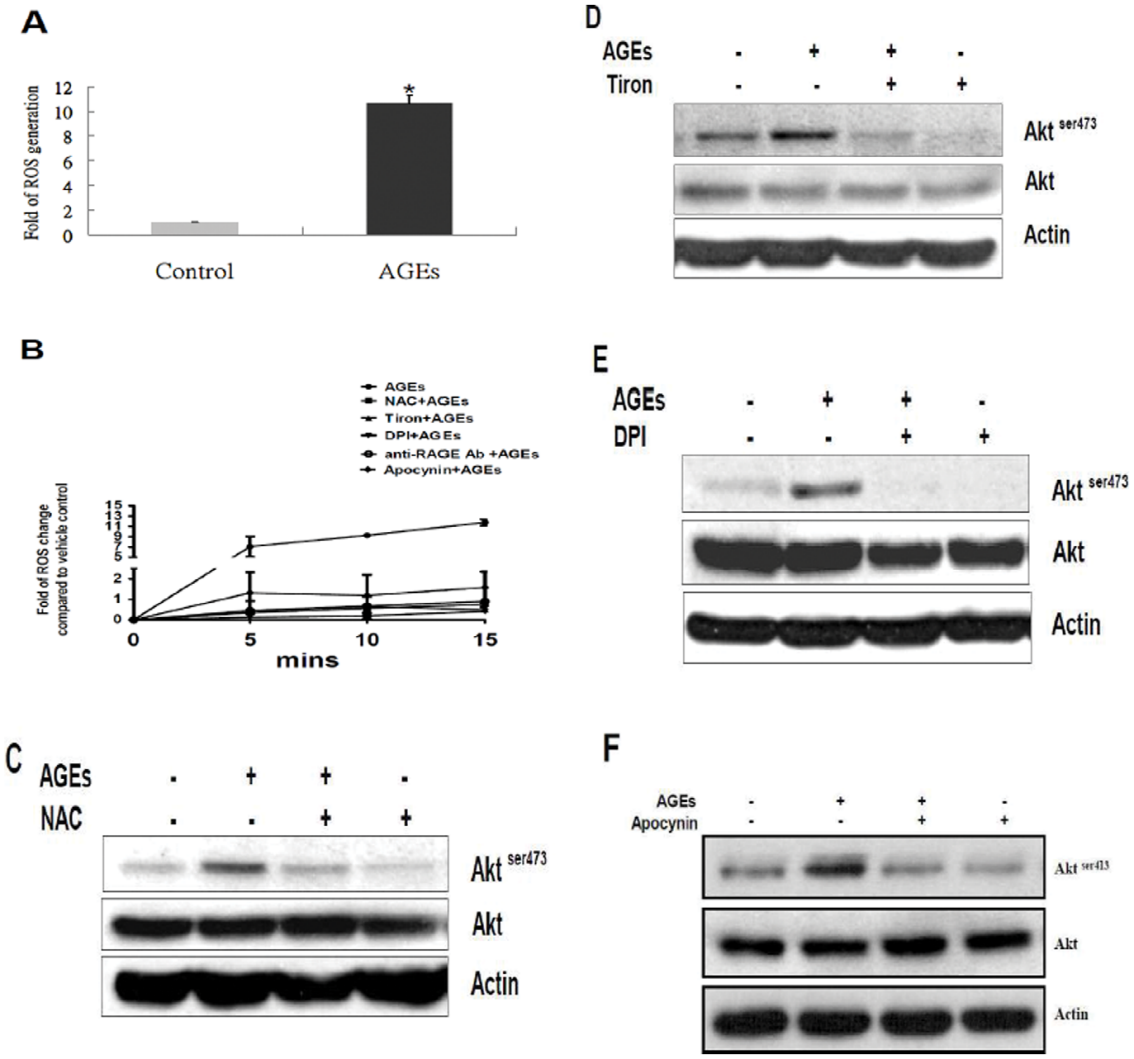
The effect of NAC, Tiron, DPI and apocynin on AGEs-stimulated Akt activation in 3T3-L1 cells. (**A**) Cells were exposed to 100 µg/ml AGEs for 15 min in the serum-free medium. After treatment, cells were incubated with 20 µM CM-H2DCFDA for 30 min at 37°C. The ROS production was determined by a fluorescence reader (excitation/emission: 485/520 nm). The data represent mean ± the standard error (SE) of results from three independent experiments. Increases in the AGEs-induced ROS were statistically significant at this time point. (*: *P*<0.05) (B) After 18 hr serum starvation, 3T3-L1 cells were treated with 2 mM NAC, 2 mM Tiron, 50 µM DPI, 25 µM apocynin, or RAGE antibodies (1 µg/ml) for 60 min, and then ROS production was determined by a fluorescence reader (excitation/emission: 485/520 nm). After 18 hr serum starvation, 3T3-L1 cells were treated with 2 mM NAC (**C**), 2 mM Tiron (**D**), 50 µ M DPI (**E**), or 25 µM apocynin (**F**) for 60 min, and then challenged with 100 µg/ml AGEs for 15 min. Cell lysates were immunoblotted with antibodies specific for phospho-Akt (Akt^ser473^), Akt or actin. Data are representative of three independent experiments yielding similar results.

It has been reported that the AGEs-RAGE signaling triggers ROS production [8]–[13]. Furthermore, ROS have been shown to be required for AGEs-stimulated PI3K activation in renal mesangial cells [10]. To examine whether AGEs stimulate ROS generation in 3T3-L1 cells, we challenged cells with 100 µg/ml AGEs for 15 min. Fig. 2A showed that ROS were induced by AGEs in 3T3-L1 cells. We then examined whether ROS are responsible for the activation of Akt by AGEs in 3T3-L1 cells. 3T3-L1 cells were treated with 2 mM N-acetyl-cysteine (NAC, a ROS scavenger), 2 mM Tiron (a superoxide scavenger) or NADPH oxidase inhibitors (50 µM DPI or 25 µM apocynin) for 60 min, and then challenged with 100 µg/ml AGEs for 15 min. Addition of NAC, Tiron, DPI or apocynin attenuated AGEs-stimulated ROS generation (Fig. 2B). The RAGE blocking antibodies also decreased AGEs-stimulated ROS generation (Fig. 2B), suggesting the involvement of RAGE in the generation of ROS by AGEs. Pretreatment of 3T3-L1 cells with NAC (Fig. 2C) or Tiron (Fig. 2D) suppressed the AGEs-stimulated Akt phosphorylation, suggesting the involvement of superoxide in the activation of Akt by AGEs. Furthermore, pretreatment of 3T3-L1 cells with DPI (Fig. 2E) or 25 µM apocynin (Fig. 2F), another NADPH oxidase inhibitor, blocked Akt activation stimulated by AGEs. These results suggest that AGEs activate Akt via superoxide generated by NADPH oxidase in 3T3-L1 cells.

### Protein Tyrosine Kinase Src, a ROS Target, is Involved in AGEs-mediated Akt Activation in 3T3-L1 Cells

**Figure 3:**
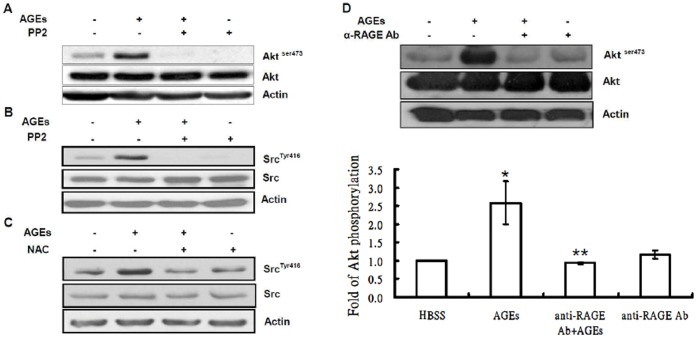
Involvement of Src and RAGE in AGEs-stimulated Akt activation in 3T3-L1 cells. (**A**)*:* Serum-starved quiescent 3T3-L1 cells were pretreated with and without 10 µM PP2 for 30 min, and then challenged with 100 µg/ml AGEs for 15 min. Total cell lysates were immunoblotted with antibodies specific for phospho-Akt (Akt^ser473^), Akt or actin. Serum-depleted cells were pretreated with and without 10 µM PP2 for 30 min (**B**), 2 mM NAC (**C**) or RAGE antibodies (1 µg/ml) (**D**) for 60 min, and then challenged with 100 µg/ml AGEs for 15 min. Total cell lysates were immunoblotted with antibodies specific for phospho-Src (Src^Tyr416^), total Src (Src), Akt, phospho-Akt (Akt^ser473^) or actin. Data are representative of three independent experiments yielding similar results.

RAGE has been shown to associate with and activate protein tyrosine kinase Src in endothelial, vascular smooth muscle and skeletal muscle cells [25]–[27]. We examined whether Src is involved in the activation of Akt by AGEs in 3T3-L1 cells utilizing PP2, a Src tyrosine kinase inhibitor. Fig. 3A shows that pretreatment of 3T3-L1 cells with 25 µM PP2 abolished the AGEs-stimulated Akt activity. Phosphorylation of Src at Tyr 416 in the activation loop of the kinase domain elevates the kinase activity of Src. We then further examined whether AGEs activate Src employing Western blot analysis with phospho-Src (pY416) antibodies. As shown in Fig. 3B, AGEs enhanced tyrosine phosphorylation levels of Src, which were significantly blocked by PP2. These results suggested that Src is activated by AGEs and involved in AGEs-mediated Akt activation in 3T3-L1 cells.

Src has been shown to be activated by reactive oxygen species in NIH3T3 cells [28], [29]. We next investigated whether Src links reactive oxygen species production to Akt activation in the AGEs-elicited signaling pathway. 3T3-L1 cells were pretreated with 2 mM NAC for 60 min, and then challenged with 100 µg/ml AGEs for 15 min. Fig. 3C showed that NAC inhibited AGEs-induced tyrosine phosphorylation of Src. These results suggest that Src is downstream of NAD(P)H oxidase, but upstream of Akt in the AGEs-stimulated signaling pathway.

### AGEs Activate Akt via RAGE in 3T3-L1 Cells

RAGE is the receptor that mediates the effects of AGEs on the activation of ERK1/2 and PI3-kinase [1], [3], [5], [10], [24]. The binding site for AGEs is located on the V-type immunoglobulin domain of RAGE. We examined whether the AGEs-stimulated Akt activation is mediated by RAGE using RAGE neutralizing antibodies that recognized the amino acid residues on the V-type domain of RAGE. 3T3-L1 cells were pretreated with and without 10 µg/ml anti-RAGE antibody for 60 min, and then challenged with 100 µg/ml AGEs for 15 min. Fig. 3D showed that Akt activation stimulated by AGEs was abolished by anti-RAGE antibodies, suggesting that RAGE mediates the activation of Akt by AGEs in 3T3-L1 cells.

### AGEs Activate Akt via the Src-mediated IGF-1R Transactivation

**Figure 4:**
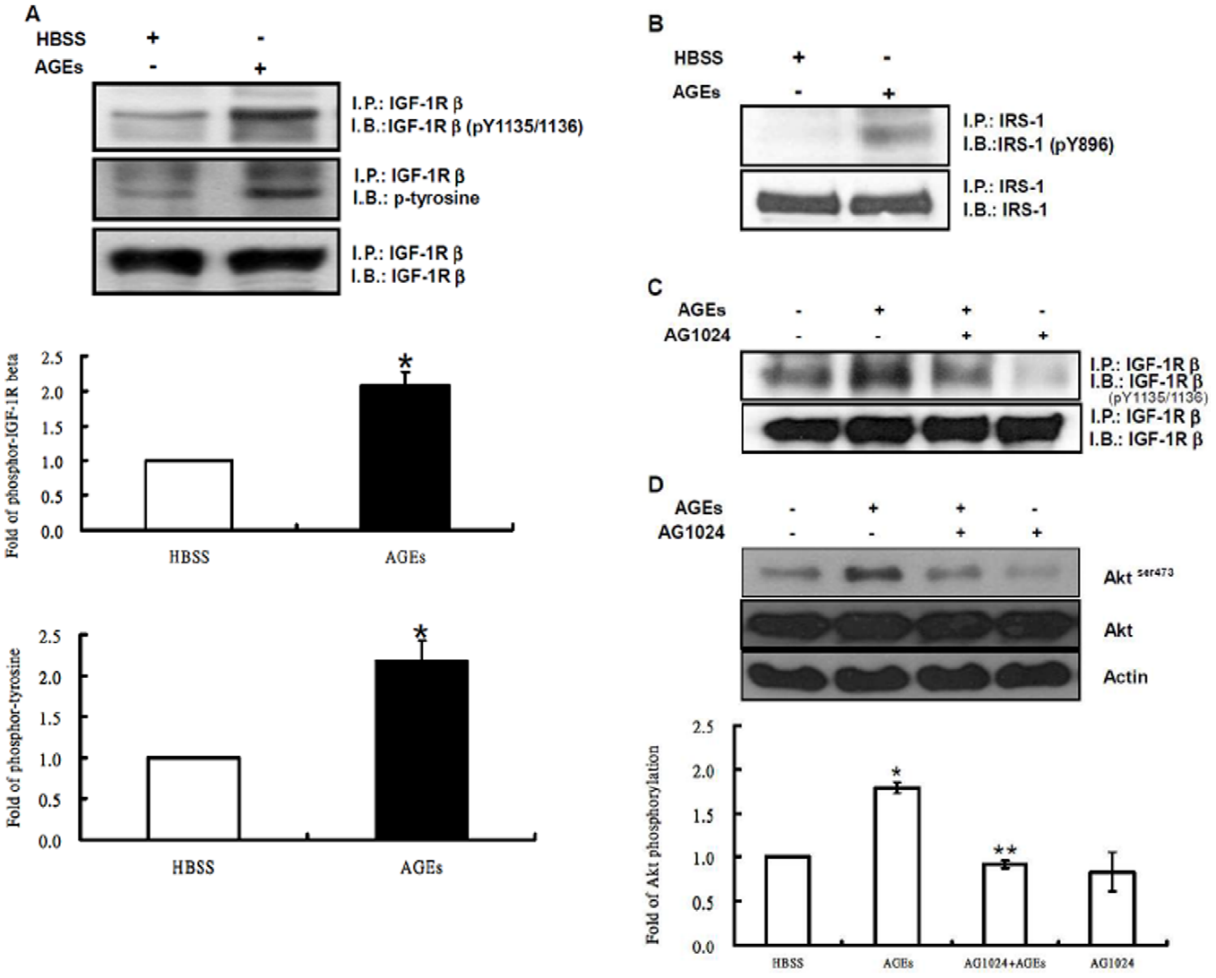
Transactivation of IGF-1R by AGEs in 3T3-L1 cells. Serum-depleted 3T3-L1 cells were stimulated with 100 µg/ml AGEs for 15 min, and cell lysates were immunoprecipitated with IGF-1R (**A**) or IRS-1 (**B**) antibodies followed by Western blotting with phospho-tyrosine, phospho-IGF-1Rβ(IGF-1Rβ^pY1135/1136^), phospho-IGF-1R (IGF-1Rβ^pY1131^), total IGF-1Rβ (IGF-1Rβ), IRS-1 or actin antibodies. Serum-depleted 3T3-L1 cells were pretreated with and without 2 µM AG1024 for 30 min and then challenged with 100 µg/ml AGEs for 15 min. Cell lysates were immunoprecipitated with IGF-1Rβsubunit (IGF-1β) antibody followed by immunoblotting with phospho-IGF-1Rβ (IGF-1Rβ^pY1135/1136^) and IGF-1Rβ(**C**). Total cell lysates were also immunoblotted with antibodies specific for phospho-Akt (Akt^ser473^) or Akt (**D**). Data are representative of three independent experiments yielding similar results.

It has been shown that IGF-1 receptor cross-talks with several other receptors such as angiotensin II, endothelin-1, estrogen or EGF [30]–[36]. We examined whether IGF-1 receptor is involved in AGEs-mediated Akt activation in 3T3-L1 cells. Cultured cells were stimulated with 100 µg/ml AGEs for 15 min, and IGF-1 receptor βsubunit (IGF-1Rβ) in the cell lysates was immunoprecipitated with its antibody. The immunoprecipitates were subjected to Western blotting with phospho-tyrosine or phospho-IGF-1Rβ (p-Y1131 or p-Y1135/1136) antibodies. Fig. 4A showed that AGEs increased total tyrosine phosphorylation levels of IGF-1Rβand phosphorylation levels of Tyr1135/1136 on IGF-1Rβ. However, the tyrosine phosphorylation levels of Tyr1131 were not significantly affected by AGEs (data not shown). Both immunoprecipitation and Western blot analysis experiments revealed that AGEs also increased tyrosine phosphorylation levels of IRS-1 (Fig. 4B). Pretreatment of 3T3-L1 cells with AG1024, an IGF-1 receptor kinase inhibitor, attenuated the phosphorylation levels of tyrosine 1135/1136 on IGF-1R β-subunit induced by AGEs (Fig. 4C) and attenuated the activation of Akt (Fig. 4D). These results suggest that AGEs activate Akt signaling by transactivating IGF-1R in 3T3-L1 cells.

**Figure 5:**
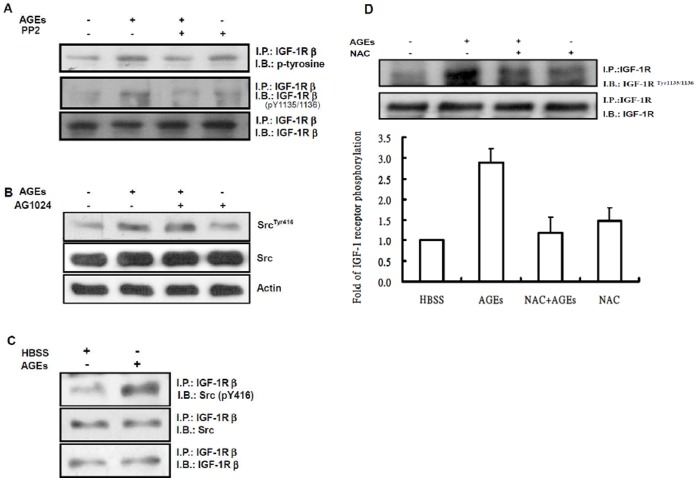
Transactivation of IGF-1R by AGEs is mediated by Src in 3T3-L1 cells. (**A**) Serum-depleted 3T3-L1 cells were pretreated with and without 10 µM PP2 for 30 min and then challenged with 100 µg/ml AGEs for 15 min. Cell lysates were immunoprecipitated with IGF-1Rβantibody followed by immunoblotting with phospho-tyrosine, IGF-1Rβ or phospho-IGF-1Rβ (IGF-1Rβ^pY1135/1136^) antibodies. (**B**) Total lysates from cells treated with and without 2 µM AG1024 and then challenged with 100 µg/ml AGEs for 15 min were subjected to Western blotting with antibodies specific for phospho-Src (Src^Tyr416^), Src or actin antibodies. (**C**) Total lysates from cells treated with and without 100 µg/ml AGEs were immunoprecipitated with the IGF-1Rβantibody followed by immunoblotting with IGF-1Rβ, Src and p-Src antibodies. (**D**) Serum-starved 3T3-L1 cells were pretreated with and without 2 mM NAC for 60 min and then challenged with 100 µg/ml AGEs for 15 min. Cell lysates were immunoprecipitated with IGF-1Rβantibody followed by immunoblotting with phospho-tyrosine or phospho-IGF-1Rβ (IGF-1Rβ^pY1135/1136^) antibodies. The data represent mean ± the standard error (SE) of results from three independent experiments. The densitometrical data were shown as the means ± SEM of three independent experiments. *P<0.05 compared with control group and **P<0.05 compared with AGEs group.

We next investigated whether Src is upstream of IGF-1 receptor. 3T3-L1 cells were pretreated with and without 10 µM PP2 for 30 min, and then incubated with 100 µg/ml AGEs for 15 min. After incubation, IGF-1Rβproteins in the cell lysates were immunoprecipitated with its antibody. The immunocomplexes were subjected to Western blotting with phospho-tyrosine or phospho-IGF-1Rβ (p-Y1135/1136) antibodies. Fig. 5A showed that addition of PP2 attenuated AGEs-stimulated tyrosine phosphorylation of IGF-1Rβ. In contrast, pretreatment of 3T3-L1 cells with AG1024 did not affect AGEs-stimulated Src kinase activity (Fig. 5B).

We then examined whether Src associates with IGF-1Rβ in 3T3-L1 cells. Serum-depleted 3T3-L1 cells were stimulated with 100 µg/ml AGEs for 15 min, and IGF-1Rβ and its interacting proteins were immunoprecipitated with IGF-1Rβ antibody. The immunocomplexes were subjected to Western blot analysis with IGF-1Rβ, Src and phospho-Src (p-Src) antibodies. The results revealed that Src associated with IGF-1Rβ and AGEs enhanced the association of p-Src with IGF-1Rβ (Fig. 5C). Moreover, pretreatment of 3T3-L1 cells with 2 mM NAC, and then stimulated with 100 µg/ml AGEs for 15 min attenuated the phosphorylation levels of tyrosine 1135/1136 on IGF-1Rβ induced by AGEs (Fig. 5D). These results suggest that ROS and Src are upstream of IGF-1 receptor, and that Src phosphorylates IGF-1Rβ.

### Phospho-Cav-1 is Positively Involved in AGEs-mediated Akt Activation in 3T3-L1 Cells

**Figure 6:**
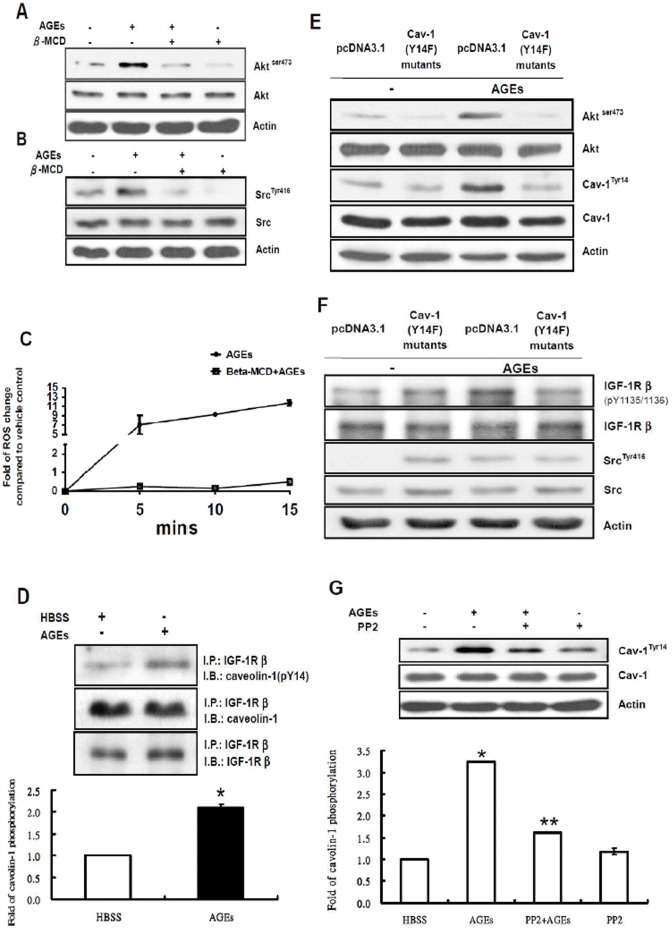
Involvement of phospho-Cav-1 in AGEs-mediated Akt activation in 3T3-L1 cells. (**A**) Serum-depleted 3T3-L1 cells were pretreated with and without 50 µM β-MCD for 60 min and then challenged with 100 µg/ml AGEs for 15 min. Total cell lysates were immunoblotted with antibodies specific for phospho-Akt (Akt^ser473^), Akt or actin. Data are representative of three independent experiments yielding similar results. (**B**) Serum-depleted 3T3-L1 cells were pretreated with and without 20 µM β-MCD for 60 min and then challenged with 100 µg/ml AGEs for 15 min. Cell lysates were subjected to Western blotting with antibodies specific for phospho-Src (Src^Tyr416^), total Src (Src) or actin antibodies. (**C**) Serum-depleted 3T3-L1 cells were pretreated with and without 50 µM β-MCD for 60 min and then challenged with 100 µg/ml AGEs for 15 min. After treatment, cells were incubated with 20 µM CM-H2DCFDA for 30 min at 37°C. The ROS production was determined by a fluorescence reader (excitation/emission: 485/520 nm). The data represent mean ± the standard error (SE) of results from three independent experiments. (**D**) Serum-depleted 3T3-L1 cells were stimulated with 100 µg/ml AGEs for 15 min, and cell lysates were immunoprecipitated with IGF-1Rβ antibody followed by immunoblotting with phospho-Cav-1 (Cav-1 ^tyr14^), Cav-1 or IGF-1Rβ. (**E**) Cell lysates from vector control and cells expressing Cav-1 Y14F were subjected to Western blotting with phospho-Cav-1 (Cav-1 ^tyr14^), Cav-1, phospho-Akt or Akt. (**F**) Cell lysates from vector control and cells expressing Cav-1 Y14F were subjected to Western blotting with phospho-Src (Src^Tyr416^), Src or actin antibodies. Cell lysates were also immunoprecipitated with IGF-1Rβantibody followed by immunoblotting with phospho-IGF-1Rβ (IGF-1Rβ^pY1135/1136^) or IGF-1Rβ. (**G**) Serum-depleted cells were pretreated with and without 10 µM PP2 for 30 min and then challenged with 100 µg/ml AGEs for 15 min. Total cell lysates were immunoblotted with antibodies specific for phospho-caveolin-1 (Cav-1 ^tyr14^), Cav-1 or actin. Data are representative of three independent experiments yielding similar results. *, statistically significant differences (* and **, P<0.05 *versus* control).

Caveolae are involved in the regulation of intracellular signaling. RAGE and several tyrosine kinase receptors including IGF-1 receptors (IGF-1R) are localized in caveolae or lipid raft [15]–[18]. Caveolae are principally composed of cholesterol and sphingolipids. The cholesterol-binding reagent, β-methylcyclodextrin (β-MCD) is widely used to deplete cholesterol and disrupt caveolae structures. To examine whether caveolae are involved in AGEs-mediated Akt activation, we pre-incubated 3T3-L1 cells with 50 µM β-MCD for 60 min, and then challenged cells with 100 µg/ml AGEs for another 15 min. As shown in Fig. 6A and B, disruption of caveolae by β-MCD inhibited Akt and Src activation by AGEs. There are several NADPH oxidase isoforms (Nox1-Nox5). It has been shown that Nox1, Nox2 and Nox4 are located in caveolae [37]. Addition of β-MCD has been shown to inhibit ROS generation in several cell types [38]–[40]. Similarly, disruption of caveolae by β-MCD also decreased AGEs-stimulated ROS production in 3T3-L1 cells (Fig. 6C). These results suggest that the intact structure or components of caveolae is essential in AGEs-mediated Akt activation.

We further investigated whether Cav-1, the major component in caveolae, is involved in the AGEs signaling cascade. It has been reported that IGF-1 receptor directly interacts with Cav-1 in 3T3-L1 cells [41]. Therefore, we examined whether Cav-1 is involved in the AGEs-mediated IGF-1R transactivation and Akt activation employing co-immunoprecipitation followed by Western blot analysis. 3T3-L1 cells were incubated with and without 100 µg/ml AGEs for 15 min. After incubation, IGF-1R proteins were immunoprecipitated by IGF-1Rβ antibodies, and the immunoprecipitates were subjected to Western blot analysis using Cav-1 and phospho-Cav-1 (at Y14) antibodies. Fig. 6D showed that Cav-1 associated with the β-subunit of IGF-1R, and that addition of AGEs enhanced the association of phospho-Cav-1 with IGF-1Rβ (Fig. 6D). To examine the role of tyrosine phosphorylation of Cav-1, we generated stable cell lines expressing vector pcDNA3.1 or Cav-1 Y14F mutant, and then challenged both vector control and Cav-1 Y14F expressing cells with 100 µg/ml AGEs for 15 min. The cell lysates were then subjected to Western blot analysis with Cav-1, p-Cav-1, Akt, p-Akt, Src, p-Src, pIGF-1Rβ^pY1131^ and IGF-1Rβ antibodies. The results showed that Cav-1 Y14F expressing cells exhibited reduced p-Cav-1 and phosphor-Akt levels in the absence of AGEs (Fig. 6E). Cav-1 Y14F expressing cells exhibited slightly elevated p-IGF-1R and phospho-Src levels in the absence of AGEs (Fig. 6F). However, unlike control cells, addition of AGEs failed to phosphorylate Y14 on Cav-1, phosphorylate Y1135/1136 on IGF-1R, phosphorylate Y416 on Src and to activate Akt in Cav-1 expressing cells (Fig. 6F), suggesting that tyrosine phosphorylation of Cav-1 is essential for AGEs-mediated Akt activation.

Src is known to be present in caveolae and phosphorylate Cav-1 [26]. To determine whether Src is responsible for the enhanced tyrosine phosphorylation of Cav-1, we incubated 3T3-L1 cells with and without 10 µM PP2 for 30 min, and then challenged cells with 100 µg/ml AGEs for 15 min. Cell lysates were then subjected to Western blot analysis with p-Cav-1 antibodies. Treatment of 3T3-L1 cells with AGEs increased the tyrosine phosphorylation levels of Cav-1, whereas addition of PP2 decreased the AGEs-enhanced tyrosine-phosphorylated Cav-1 levels (Fig. 6G). These results suggest that Src is responsible for the AGEs-stimulated tyrosine phosphorylation of Cav-1.

### AGEs Promote Adipogenic Differentiation of 3T3-L1 Preadipocytes

**Figure 7:**
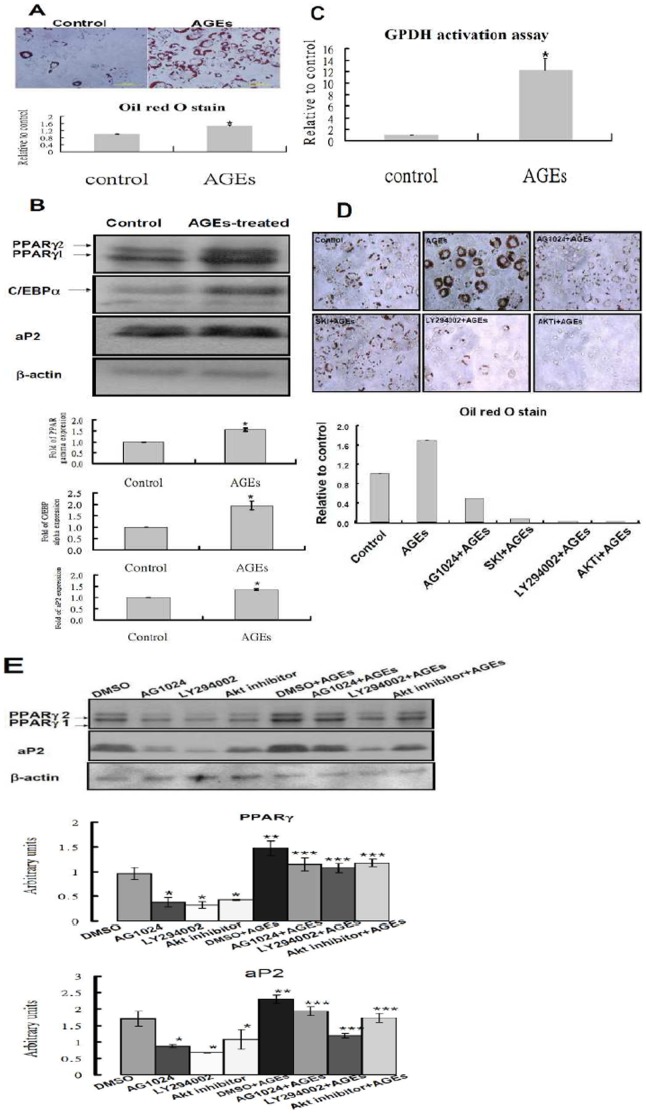
Promotion of adipogenesis of 3T3-L1 cells by AGEs-treatment. (**A**) 3T3-L1 cells were treated with and without 100 µg/ml AGEs during differentiation.The adipogenic induction medium contains 0.5 mM isobutylmethylxanthine, 1 mM dexamethasone, and 1.7 mM insulin. At day 12, cells were subjected to the Oil Red O staining. The oil droplet contents were quantified by measuring the OD510 nm. (*, *P*<0.05 *versus* control) (**B**) 3T3-L1 cells were treated with and without 100 µg/ml AGEs during differentiation. At day 5, cell lysates were subjected to Western blot analysis using the PPARγ, C/EBPα, aP2 and the loading control actin antibodies. (**C**) Cells from 3T3-L1 and 100 µg/ml AGEs-treated cells were harvested on day 8 after adipogenic induction and were subjected to GPDH activity assay by reading the absorbance of NADH at 340 nm. The results are the means ± SEM of three independent experiments. (*, *P*<0.05 *vs.* control) (**D**) 3T3-L1 cells were treated with and without 100 µg/ml AGEs in the presence of and absence of 10 µM AG1024, 15 µM LY294002, or 5 µM Akt inhibitor during differentiation. Four days after adipogenic induction, cells were subjected to the Oil Red O staining and the oil droplet contents were quantified by measuring the OD510 nm. (E) 3T3-L1 cells were treated with and without 100 µg/ml AGEs in the presence of and absence of 10 µM AG1024, 15 µM LY294002, or 5 µM Akt inhibitor during differentiation. The concentration of DMSO is 0.05% for control and inhibitor-treated groups. At day 5, cell lysates were subjected to Western blot analysis using PPARγ, aP2 and actin antibodies. (*, *P*<0.05 DMSO *versus* inhibitors; **, *P*<0.05 DMSO vs. DMSO+AGEs, ***, P<0.05 DMSO+AGEs vs. inhibitors+AGEs).

Treatment of 3T3-L1 cells with AGEs has been shown to accelerate lipid droplet formation based only on the Oil Red O staining [42]. We re-examined the effect of AGEs on the differentiation of 3T3-L1 cells. Fig. 7A showed that AGEs promoted the differentiation of 3T3-L1 cells based on staining and quantization of the oil droplets. PPARγ and C/EBPα are the master transcription factors governing the adipocyte differentiation [43]. Fig. 7B showed that levels of PPARγand C/EBPα were elevated in AGEs-treated cells as compared to those in untreated 3T3-L1 cells. AGEs treatment also increased levels of markers for differentiated adipocytes, aP2 (Fig. 7B) and GPDH activity (Fig. 7C). In contrast, addition of AG1024, LY 294002 or Akt inhibitor attenuated the differentiation of 3T3-L1 cells and the promoting effect of AGEs on adipogenesis (Fig. 7D). Consistently, PPARγ and aP2 levels that are up-regulated during adipogenesis and AGEs-enhanced adipogenesis were attenuated by AG1024, LY 294002 and Akt inhibitor (Fig. 7E).

## Discussion

Chronic hyperglycemia which is characteristic of diabetes facilitates the formation of AGEs [1]–[6]. Formation of AGEs is considered a potential link between hyperglycemia and chronic diabetic complications such as diabetic nephropathy and retinopathy [1], [4]–[6]. AGEs also accumulate in many different tissues during the normal aging process [1]–[3]. Many of the effects of AGEs are mediated by their signaling receptor RAGE [7]. Although AGEs and RAGE have been shown to activate a variety of protein kinases such as Akt, ERK1/2, JNK and p38 MAPK, little is known about the proximal signaling events downstream of RAGE. In the present study, we showed for the first time that via RAGE, NAD(P)H oxidase and Src, AGEs transactivate IGF-1 receptor and the downstream PI3-kinase-PDK1-Akt pathway in 3T3-L1 cells. We have also found that phospho-Cav-1 plays a positively regulatory role in AGEs-mediated Akt activation.

The signaling events that link RAGE to NAD(P)H oxidase, Src and Akt remain unclear. Our results showed that AGEs activated Akt via PI3-kinase since the AGEs-stimulated Akt activity was inhibited by a PI3-K inhibitor LY294002. The effects of AGEs on Akt activation are mediated by their receptor RAGE as neutralizing RAGE antibodies attenuated the activation of Akt by AGEs. The AGEs-stimulated Akt activity was blocked by NAC, an antioxidant, Tiron, a superoxide scavenger and DPI and apocynin, NADPH oxidase inhibitor, suggesting that NAD(P)H oxidase and reactive oxygen species participate in the activation of Akt by AGEs. Src is a redox-sensitive tyrosine kinase [28], [29]. Our data showed that AGEs activated Src, and that the AGEs-stimulated Akt activation was attenuated by PP2, a Src inhibitor, suggesting the involvement of Src in the activation of Akt by AGEs. Furthermore, we found that AGEs-stimulated Src kinase activity was blocked by NAC, suggesting that Src is downstream of NAD(P)H oxidase. These results suggest that AGEs activate NAD(P)H oxidase and produce reactive oxygen species which then stimulate Src kinase activity leading to activation of the PI3-kinase-PDK1-Akt pathway.

Binding of IGF-1 to IGF-1R is known to activate the downstream Akt and ERK1/2 by tyrosine-phosphorylating insulin receptor substrate proteins (IRS) and Shc. Besides stimulating its own signaling pathways, IGF-1 receptor can be transactivated by a variety of receptors such as angiotensin II, endothelin-1, estrogen, and epidermal growth factor [30]-[36]. IGF-1 receptor is ubiquitously expressed and also present in 3T3-L1 cells [41]. Several lines of evidence suggest the involvement of IGF-1 receptor in the activation of Akt by AGEs in 3T3-L1 cells. First, AGEs induced tyrosine phosphorylation of IGF-1Rβ and the downstream IRS-1. Second, addition of IGF-1 receptor kinase inhibitor AG1024 attenuated AGEs-stimulated Akt activity. Third, AGEs activated Src and enhanced the association of activated Src with IGF-1Rβ. Furthermore, inhibition of Src with PP2 blocked both AGEs-stimulated tyrosine phosphorylation of IGF-1Rβand AGEs-stimulated Akt activity. These results suggest that Src is upstream of IGF-1 receptor. Precedents for the involvement of Src in the transactivation of IGF-1 receptor were reported by Zahradka *et al* [34] and Bouallegue *et al* [35]. Both groups found that Ang II or endothelin-1 induces the tyrosine phosphorylation levels of IGF-1Rβ and a Src inhibitor PP1 or PP2 weakens the phosphorylation of IGF-1 receptor. Src is also a mediator of IGF-1 receptor transactivation in response to EGF [36]. Thus, activation of Src appears to be essential for the transactivation of IGF-1 receptor by Ang II, endothelin-1, EGF and AGEs.

Although Ang II, EGF and endothelin-1 are reported to enhance tyrosine phosphorylation levels of IGF-1Rβ, the identity of the phosphorylated tyrosine residues remains unknown [30]–[36]. Y1131, Y1135 and Y1136 are the autophosphorylation sites on the activation loop of the IGF-1Rβ catalytic domain [44], [45]. Binding of IGF1 to the α subunit of IGF-1 receptor triggers a conformational change leading to autophosphorylation of these tyrosine residues. It has been shown that the first site of autophosphorylation is predominantly Y1135, followed by Y1131 and then by Y1136, and that each phosphorylation increases receptor tyrosine kinase activity [45]. We examined whether these tyrosine residues on IGF-1Rβ are phosphorylated by AGEs using antibodies recognizing p-Y1135/1136 and p-Y1131 on IGF-1Rβ. The results indicated that the phosphorylation levels of Y1135/1136, but not Y1131, on IGF-1Rβ were enhanced by AGEs. These results indicate that AGEs triggers the phosphorylation of at least Y1135 and Y1136 on IGF-1Rβ. Src has been shown to phosphorylate Y1131, Y1135 and Y1136 on IGF-1R and activate IGF-1 receptor [46], [47]. Consistently with those reports, our data showed that AGEs activated Src and induced phosphorylation of Y1135 and Y1136 on IGF-1Rβ. Blockage of Src kinase activity with PP2 attenuated the AGEs-induced phosphorylation of Y1135 and Y1136 on IGF-1 receptor and AGEs-stimulated Akt activation. Therefore, Src likely mediates the effects of AGEs on the tyrosine phosphorylation and activation of IGF-1 receptor.

Many signaling proteins and receptors are enriched in caveolae. Previous studies showed that RAGE, IGF-IR, and Src family tyrosine kinases localize in caveolae [13]–[18]. Cav-1 has been shown to positively regulate IGF-1 signaling by stabilizing IGF-1 receptor and IRS-1. Protein levels of IRS-1 and IGF-1 receptor are reduced in Cav-1^−/−^ mouse embryo fibroblasts [48]. Reduced IRS-1 levels were also reported in Cav-1 knock-down H9C2 cells [49]. In contrast, over-expression of Cav-1 significantly increases protein levels of IGF-1 receptor in MCF-7 breast cancer cells [50]. However, in contrast to mouse embryo fibroblasts and H9C2 cells, knock-out or knock-down of Cav-1 does not affect the IGF-1 signaling in 3T3-L1 cells [51], [52]. It appears that the role of Cav-1 in IGF-1 signaling is cell type-specific. In this study, our data showed that depletion of cholesterol by β-MCD significantly reduced AGEs-stimulated Akt activation in 3T3-L1 cells, suggesting that caveolae and Cav-1 are important for AGEs-mediated Akt activation. IGF-1 receptor has been shown to directly interact with Cav-1 in 3T3-L1 cells [41]. Consistently with these findings, our data showed that Cav-1 associated with IGF-1 receptor in 3T3-L1 cells. Cav-1 can be phosphorylated on Tyr 14 by Src family kinases [26], [53]. We further found that addition of AGEs activated Src and increased the phosphorylation of Tyr 14 on Cav-1 (p-Cav-1). AGEs also increased the association of p-Cav-1 with IGF-1 receptor in 3T3-L1 cells. In contrast, addition of PP2 attenuated the tyrosine phosphorylation state of Cav-1 and IGF-1Rβ and decreased the downstream Akt signaling. Furthermore, expression of Cav-1 Y14F decreased the levels of p-Cav-1 and AGEs-stimulated Akt activation. These results suggest that p-Cav-1 positively regulates the transactivation of IGF-1 receptor by RAGE and Src.

3T3-L1 cells can differentiate into adipocytes. The IGF-1 receptor signaling is required for the differentiation of 3T3-L1 preadipocytes [54]. Since AGEs transactivate IGF-1 receptor, we examined the effects of AGEs on the differentiation of 3T3-L1 cells, and found that AGEs treatment increased the amount of lipid droplet accumulation as compared to the control. Similar results have been reported by other investigators [42]. PPARγ is the master transcription factor governing adipogenesis [43]. Ectopic expression of PPARγ in fibroblasts induces adipogenesis [55]. In contrast, embryonic fibroblasts derived from PPARγ-null mice fail to differentiate into adipocytes [56]. C/EBPα is another important transcription factor regulating adipogenesis. Ectopic expression of the C/EBPα has been shown to promote adipogenesis [57]. We further showed that AGEs treatment increased levels of PPARγand C/EBPα. AGEs treatment also increased levels of the adipocyte markers aP2 and GPDH activity. These results suggest that AGEs promote adipogenesis by up-regulating PPARγand C/EBPα. Furthermore, addition of AG1024, LY 294002 or Akt inhibitor attenuated the level of PPARγ and aP2 and the promoting effect of AGEs on the differentiation of 3T3-L1 cells, suggesting that IGF-1 receptor, PI3-Kinase and Akt are involved in the facilitation of adipogenesis by AGEs. Obesity is known to increase the risk of developing type II diabetes. In the cell level, obesity is due to increased differentiation of preadipocytes and enlargement of existing adipocytes. It is known that AGEs formation is enhanced in diabetes. The fact that AGEs promote adipogenesis (i.e., obesity) might further accelerate diabetes and create a vicious cycle. Further investigation is required to delineate the complicated interactions among AGEs, obesity and diabetes.

**Figure 8:**
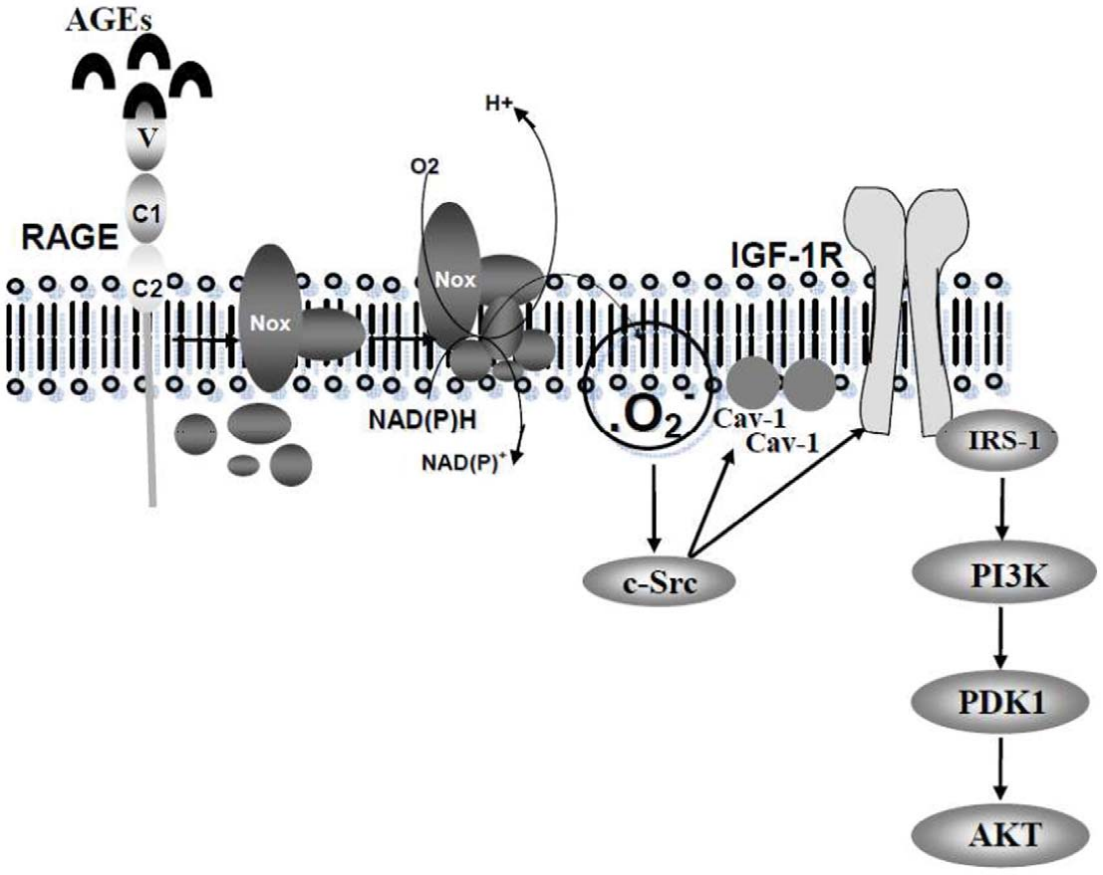
A proposed model for the activation of Akt by AGEs in 3T3-L1 cells. AGEs, via RAGE, activate NAD(P)H oxidase and produce reactive oxygen species (ROS) which then stimulate Src kinase activity. Src subsequently phosphorylates and activates IGF-1 receptor which acts as a stimulator for PI3-kinase, PDK-1 and Akt. Src also phosphorylates caveolin-1 and enhances IGF-1 receptor-mediated signaling.

In summary, the data presented in this study suggest that IGF-1 receptor transactivation is required for the activation of the PI3-kinase-Akt pathway by AGEs in 3T3-L1 cells. This mechanism involves RAGE that couples to NAD(P)H oxidase to stimulate Src. The activated Src in turn phosphorylates and activates IGF-1 receptor and its downstream PI3-kinase-Akt pathway. Our data also reveal that Src phosphorylates Cav-1, and that p-Cav-1 associates with IGF-1 receptor and is positively involved in the activation of the PI3-kinase-Akt pathway by AGEs (Fig. 8). Our study shows that besides G protein-coupled receptors (Angiotensin II and endothelin-1 receptors) and nuclear receptors (estrogen receptors), RAGE, a receptor of the immunoglobulin-like superfamily, can also transactivate IGF-1 receptor. Since AGEs and RAGE are involved in diabetic complications and other diseases, IGF-1 receptor antagonists and cholesterol depleting agents might have therapeutic application to these disorders.
